# *KRAS*, *GNAS*, and *RNF43* mutations in intraductal papillary mucinous neoplasm of the pancreas: a meta-analysis

**DOI:** 10.1186/s40064-016-2847-4

**Published:** 2016-07-26

**Authors:** Ju-Han Lee, Younghye Kim, Jung-Woo Choi, Young-Sik Kim

**Affiliations:** Department of Pathology, Korea University Ansan Hospital, 123, Jeokgeum-ro, Danwon-gu, Ansan-si, Gyeonggi-do 425-707 Republic of Korea

**Keywords:** Intraductal papillary mucinous neoplasm, KRAS, GNAS, Meta-analysis

## Abstract

**Background:**

The prevalence and clinical significances of *KRAS*, *GNAS,* and *RNF43* mutations in patients with pancreatic intraductal papillary mucinous neoplasm (IPMN) remain elusive. To evaluate the incidence of the gene mutations and clinicopathologic differences between *KRAS* and *GNAS* mutations in pancreatic cystic lesions, we performed a meta-analysis of published 33 KRAS, 11 GNAS, and 4 RNF43 studies including 1253, 835, and 143 cases, respectively.

**Methods:**

We pooled the results of relevant studies identified using the PubMed and EMBASE databases. The effect sizes of outcome parameters were computed by the prevalence rate, weighted mean difference, or odds ratio (OR) using a random-effects model.

**Results:**

The pooled prevalence of *KRAS*, *GNAS,* and *RNF43* mutations in IPMN was 61, 56, and 23 %, respectively. The *KRAS* (OR 7.4 and 71.2) and *GNAS* (OR 30.2 and 15.3) mutations were more frequently found in IPMNs than in mucinous cystic neoplasms and in serous cystadenomas, respectively. Of the microscopic subtypes of IPMN, *KRAS* and *GNAS* were frequently mutated in gastric type (OR 2.7, *P* < 0.001) and intestinal type (OR 3.0, *P* < 0.001), respectively. *KRAS* mutation was infrequently found in high-grade dysplasia lesions of IPMN (OR 0.6, *P* = 0.032). *GNAS* mutation was associated with male (OR 1.9, *P* = 0.012).

**Conclusions:**

This meta-analysis supports that *KRAS* and *GNAS* mutations could be diagnostic markers for IPMN. In addition, the frequencies of *KRAS* and *GNAS* mutations in IPMNs are highly variable according to the microscopic duct subtypes, reflecting their independent roles in the IPMN-adenocarcinoma sequence.

**Electronic supplementary material:**

The online version of this article (doi:10.1186/s40064-016-2847-4) contains supplementary material, which is available to authorized users.

## Background

Intraductal papillary mucinous neoplasm (IPMN) of the pancreas is a mucin-producing and cystic tumour growing inside the pancreatic duct and forming papillary projections (Klöppel et al. 2014; Klöppel and Kosmahl 2001). IPMN is considered as a precursor of pancreatic adenocarcinoma and comprised of about 16–24 % of cystic pancreatic lesions (Klöppel et al. 2014; Klöppel and Kosmahl 2001). IPMN forms a multilocular cystic lesion and is difficult to distinguish from mucinous cystic neoplasm (MCN) (Klöppel et al. 2014; Klöppel and Kosmahl 2001).

Recently, genetic studies of IPMN lead to discover mutations of new genes, including *GNAS*, and *RNF43* (Macgregor-Das and Iacobuzio-Donahue 2013; Reid et al. 2014). In addition to the previously known genetic alteration such as *KRAS*, these gene mutations open a new viewpoint in the field of the molecular pathogenesis of IPMN. Nevertheless, the frequencies and clinicopathologic significances of *KRAS*, *GNAS*, and *RNF43* have not been clearly delineated. Activating *GNAS* mutation at codon 201 has been identified in IPMNs of the pancreas, which runs from 36 to 79 % (Amato et al. 2014; Hosoda et al. 2015; Ideno et al. 2015; Kanda et al. 2013; Kuboki et al. 2015; Lee et al. 2014; Siddiqui et al. 2013; Singhi et al. 2014; Takano et al. 2014; Tan et al. 2015; Wu et al. 2011b). Moreover, the wide extreme diversity of *KRAS* mutation in IPMN patients, ranged from 13 to 100 %, has been observed (Amato et al. 2014; Chadwick et al. 2009; Chang et al. 2014; Fritz et al. 2009; Furukawa et al. 2005; Hosoda et al. 2015; Ideno et al. 2015; Jang et al. 2009; Kaino et al. 1999; Kitago et al. 2004; Kobayashi et al. 2008; Kondo et al. 1997; Kuboki et al. 2015; Lee et al. 2014; Lubezky et al. 2011; Mizuno et al. 2010; Mohri et al. 2012; Mueller et al. 2003; Mulligan et al. 1999; Nakata et al. 2002; Paal et al. 1999; Raimondo et al. 2002; Schönleben et al. 2008; Sessa et al. 1994; Siddiqui et al. 2013; Singhi et al. 2014; Tada et al. 1991; Takano et al. 2014; Tan et al. 2015; Uemura et al. 2003; Wada et al. 2004; Wu et al. 2011b; Yoshizawa et al. 2002). The frequency of *RNF43* mutation was ranged from 14 to 75 % (Amato et al. 2014; Sakamoto et al. 2015; Tan et al. 2015; Wu et al. 2011a).

Therefore, in this meta-analysis, we aimed to know the exact prevalence of *KRAS, GNAS,* and *RNF43* mutations in IPMN patients, and the difference between the frequency of these mutant genes in pancreatic cystic lesions. In addition, we investigated whether *KRAS* and *GNAS* mutations have clinicopathologic significances in patients with IPMN.

## Methods

### Data collection and selection criteria

**Fig. 1:**
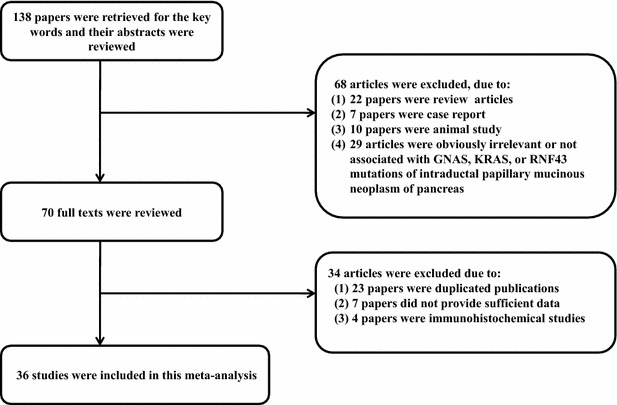
Flow diagram of article selection for this meta-analysis

We searched PubMed (http://www.ncbi.nlm.nih.gov/pubmed) and EMBASE (www.embase.com) using the keywords “KRAS”, “GNAS”, “RNF43”, “pancreas” and “intraductal papillary mucinous neoplasm”. We also manually searched the reference lists of the identified articles. Duplicate data or overlapping articles were excluded by examining the authors’ names and affiliations. Original articles reporting cases of *KRAS*, *GNAS*, and *RNF43* mutations published before June 2015 were included. When multiple articles were published by the same authors or institutions, the most recent or single informative article was selected. Articles lacking clinicopathologic data for meta-analysis, review articles without original data, conference abstracts, case reports, and articles that dealt with cell line or animal were excluded. In addition, immunohistochemical studies of *RAS* mutation were also excluded. There were no geographic or language restrictions. The selection process of the articles is shown in Fig. 1.

### Data pooling and statistics

Meta-analysis was performed as previously described (Lee et al. 2011). Briefly, effect sizes for each study were calculated by prevalence rate or odds ratio (OR) and the corresponding 95 % confidence interval (CI) using the Mantel–Haenszel method. The prevalence rate, weighted mean difference (WMD), or OR was combined using a random-effects model (DerSimonian–Laird method). Statistical heterogeneity among studies was evaluated using the Cochrane Q test and *I*^*2*^ statistics. The *I*^*2*^ statistic refers to the percentage of variation across studies that is due to heterogeneity rather than chance and does not inherently depend on the number of studies considered [*I*^*2*^ = 100 % × (Q-df)/Q]. We clarified the cutoff of *I*^*2*^ statistics for assignment of low (<25 %), moderate (25–50 %), and high (>50 %) heterogeneities. If *I*^*2*^ value was more than 50 %, subgroup analysis was done. Sensitivity analyses were performed to examine the influence of each study on the pooled prevalence rate, WMD, or OR by serially omitting an individual study and pooling the remaining studies. Publication bias was examined by funnel plots and Egger’s tests for the degree of asymmetry. Publication bias was assumed to be present if the *P* value was less than 0.1. The pooled analysis was performed using Comprehensive Meta-analysis Software version 2.0 (Biostat, Englewood, NJ, USA).

## Results

Thirty-three and eleven studies reported the frequencies of *KRAS* and *GNAS* mutations between 1253 and 835 IPMN patients, respectively (Tables 1, 2) (Amato et al. 2014; Chadwick et al. 2009; Chang et al. 2014; Fritz et al. 2009; Furukawa et al. 2005; Hosoda et al. 2015; Ideno et al. 2015; Jang et al. 2009; Kaino et al. 1999; Kanda et al. 2013; Kitago et al. 2004; Kobayashi et al. 2008; Kondo et al. 1997; Kuboki et al. 2015; Lee et al. 2014; Lubezky et al. 2011; Mizuno et al. 2010; Mohri et al. 2012; Mueller et al. 2003; Mulligan et al. 1999; Nakata et al. 2002; Paal et al. 1999; Raimondo et al. 2002; Schönleben et al. 2008; Sessa et al. 1994; Siddiqui et al. 2013; Singhi et al. 2014; Tada et al. 1991; Takano et al. 2014; Tan et al. 2015; Uemura et al. 2003; Wada et al. 2004; Wu et al. 2011b; Yoshizawa et al. 2002). On pooled analysis, *KRAS* and *GNAS* mutations were 60.9 % (95 % CI 54.3–67.1) and 55.8 % (95 % CI 48.5–62.8) in IPMN patients (Table 3). The *KRAS* and *GNAS* mutations did not differ according to the ethnicity, detection methods, and specimen type (Table 3). Additionally, eight studies presented the cases having both *KRAS* and *GNAS* mutations (Amato et al. 2014; Hosoda et al. 2015; Kuboki et al. 2015; Lee et al. 2014; Siddiqui et al. 2013; Singhi et al. 2014; Tan et al. 2015; Wu et al. 2011b). On pooled analysis, 33.5 % (95 % CI 26.2–41.6) of IPMN patients harboured both *KRAS* and *GNAS* mutations. On the other hand, four studies (Amato et al. 2014; Sakamoto et al. 2015; Tan et al. 2015; Wu et al. 2011a) described the prevalence of *RNF43* mutation among 143 IPMN patients (Additional file 1: Table S1). On pooled analysis, the frequency of *RNF43* mutation was 22.9 % (95 % CI 10.8–42.4).

**Table 1:** Characteristics of individual studies of *KRAS* mutation in patients with intraductal papillary mucinous neoplasm

Study	Country	Ethnicity	Detection method	Specimen	*KRAS *mutation (%)
Amato E	Italy	Caucasian	Sequencing	Tissue	24/48 (50.0)
Chadwick B	USA	Caucasian	Sequencing	Tissue	37/52 (71.2)
Chang X	China	Asian	Sequencing	Tissue	9/16 (56.3)
Fritz S	USA	Caucasian	Sequencing	Tissue	8/20 (40.0)
Furukawa T	Japan	Asian	ASH, sequencing	Tissue	5/6 (83.3)
Hosoda W	Japan	Asian	real-time PCR, sequencing	Tissue	59/91 (64.8)
Ideno N	Japan	Asian	HRM, sequencing	Tissue	75/95 (78.9)
Jang JY	Korea	Asian	Sequencing	Tissue	13/37 (35.1)
Kaino M	Japan	Asian	SSCP, sequencing	Pancreatic juice	12/12 (100.0)
Kitago M	Japan	Asian	Sequencing	Tissue	16/20 (80.0)
Kobayashi N	Japan	Asian	PCR/PHFA	Pancreatic juice	13/22 (59.1)
Kondo H	Japan	Asian	SSCP, sequencing	Pancreatic juice	12/13 (92.3)
Kuboki Y	Japan	Asian	Sequencing	Tissue	96/172 (55.8)
Lee LS	USA	Caucasian	Sequencing	Tissue	9/19 (47.4)
Lubezky N	Israel	Caucasian	Sequencing	Tissue	9/27 (33.3)
Mizuno O	Japan	Asian	Semiquantitative PCR	Pancreatic juice	43/53 (81.1)
Mohri D	Japan	Asian	Sequencing	Tissue	14/25 (56.0)
Muller J	Germany	Caucasian	PCR–RFLP	Tissue	4/13 (30.8)
Mulligan NJ	USA	Caucasian	PCR	Tissue	5/7 (71.4)
Nakata B	Japan	Asian	SSCP, sequencing	Tissue	19/26 (73.1)
Paal E	USA	Caucasian	Sequencing	Tissue	2/15 (13.3)
Raimondo M	USA	Caucasian	SSCP, sequencing	Tissue	29/40 (72.5)
Schönleben F	USA	Caucasian	Sequencing	Tissue	17/36 (47.2)
Sessa F	Italy	Caucasian	SSCP, sequencing	Tissue	8/26 (30.8)
Siddiqui AA	USA	Caucasian	Quantitative PCR	Cyst fluid	6/9 (66.7)
Singhi AD	USA	Caucasian	Sequencing	Cyst fluid	35/50 (70.0)
Tada M	Japan	Asian	Sequencing	Tissue	3/5 (60.0)
Takano S	Japan	Asian	Sequencing	Tissue	6/6 (100.0)
				Pancreatic juice	32/50 (64.0)
Tan MC	USA	Caucasian	Sequencing	Tissue	27/38 (71.1)
Uemura K	Japan	Asian	Sequencing	Tissue	8/10 (80.0)
Wada K	Japan	Asian	Sequencing	Tissue	15/23 (65.2)
Wu J	USA	Caucasian	PCR/ligation	Tissue	39/49 (79.6)
				Cyst fluid	68/83 (81.9)
Yoshizawa K	Japan	Asian	Sequencing	Tissue	4/7 (57.1)

*ASH* allele-specific oligonucleotide hybridization, *PCR *polymerase chain reaction, *HRM* high-resolution melt-curve analysis,* SSCP* single strand conformation polymorphism, *PHFA* preferential homoduplex formation assay, *RFLP* restriction fragment length polymorphism

**Table 2:** Characteristics of individual studies of *GNAS* mutation in patients with IPMN

Study	Country	Ethnicity	Detection method	Specimen	*GNAS* mutation (%)
Amato E	Italy	Caucasian	Sequencing	Tissue	38/48 (79.2)
Hosada W	Japan	Asian	Real-time PCR, sequencing	Tissue	55/91 (60.4)
Ideno N	Japan	Asian	HRM, sequencing	Tissue	65/110 (59.1)
Kanda M	USA	Caucasian	HRM, pyrosequencing	Pancreatic juice	49/78 (62.8)
Kuboki Y	Japan	Asian	Sequencing	Tissue	82/172 (47.7)
Lee LS	USA	Caucasian	Sequencing	Tissue	8/19 (42.1)
Siddiqui AA	USA	Caucasian	Quantitative PCR	Cyst fluid	4/9 (44.4)
Singhi AD	USA	Caucasian	Sequencing	Cyst fluid	18/50 (36.0)
Takano S	Japan	Asian	Sequencing	Tissue	4/6 (66.7)
				Pancreatic juice	34/82 (41.5)
Tan MC	USA	Caucasian	Sequencing	Tissue	23/38 (60.5)
Wu J	USA	Caucasian	PCR/ligation	Tissue	36/49 (73.5)
				Cyst fluid	51/83 (61.4)

*PCR* polymerase chain reaction, *HRM* high-resolution melt-curve analysis

**Table 3:** Prevalence of *KRAS* and *GNAS* mutations in patients with IPMN according to the ethnicity, mutation detection, and specimen type

Category	*KRAS* mutation				*GNAS* mutation			
	No. of studies	No. of cases	Prevalence (%) (95 % CI)	*P* value	No. of studies	No. of cases	Prevalence (%) (95 % CI)	*P* value
Overall	33	1253	60.9 (54.3–67.1)		11	835	55.8 (48.5–62.8)	
Ethnicity				0.106				0.429
Caucasian	15	532	55.1 (45.2–64.7)		7	374	58.4 (48.8–67.4)	
Asian	18	721	66.0 (56.9–74.0)		4	461	52.6 (41.8–63.1)	
Detection method				0.207				0.552
Sequencing	27	1017	59.0 (52.1–65.6)		9	694	54.9 (47.0–62.5)	
Non-sequencing	6	236	69.0 (54.6–80.4)		2	141	60.9 (42.3–76.9)	
Specimen type^a^				0.095				0.147
Tissue	27	929	58.9 (51.3–66.1)		8	533	61.0 (52.0–69.4)	
Cyst fluid or pancreatic juice	8	324	71.4 (58.4–81.7)		5	302	50.3 (39.1–61.5)	

*CI* confidence interval

^a^Two studies were performed in both tissue and cyst fluid

### Other cystic lesions

**Fig. 2:**
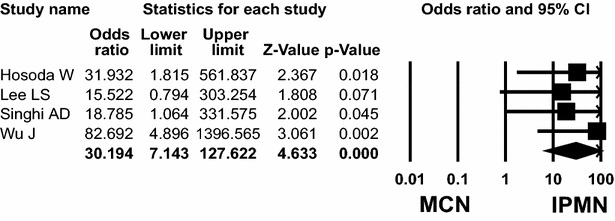
Odds ratios with corresponding 95 % confidence intervals of individual studies and pooled data for the association of *GNAS* mutation with intraductal papillary mucinous neoplasm (IPMN), compared with mucinous cystic neoplasm (MCN). Forest plot demonstrates the effect sizes and 95 % CIs for each study and overall

Seven (Hosoda et al. 2015; Lee et al. 2014; Schönleben et al. 2008; Singhi et al. 2014; Uemura et al. 2003; Wu et al. 2011b; Yoshizawa et al. 2002) and four (Hosoda et al. 2015; Lee et al. 2014; Siddiqui et al. 2013; Wu et al. 2011b) studies presented *KRAS* and *GNAS* mutations between IPMN and MCN patients. *KRAS* mutation was found in 239 (69 %) of 345 IPMN and 14 (21 %) of 67 MCN patients. *GNAS* mutation was detected in 168 (58 %) of 292 IPMN and in none of 57 MCN. The overall ORs for *KRAS* and *GNAS* mutations in IPMN patients were 7.444 (95 % CI 3.850–14.392; *P* < 0.001, Q = 4.540, *I*^*2*^ = 0.000) and 30.194 (95 % CI 7.143–127.622; *P* < 0.001, Q = 0.787, *I*^*2*^ = 0.000), compared with those mutations in MCN, respectively (Fig. 2).

Four (Hosoda et al. 2015; Lee et al. 2014; Singhi et al. 2014; Wu et al. 2011b) and five (Hosoda et al. 2015; Kanda et al. 2013; Lee et al. 2014; Singhi et al. 2014; Wu et al. 2011b) studies addressed *KRAS* and *GNAS* mutations between IPMN and serous cystadenoma (SCA) patients, respectively. *KRAS* mutation was found in 210 (72 %) of 292 IPMN and none (0 %) of 83 SCA patients. *GNAS* mutation was detected in 217 (59 %) of 370 IPMN patients and 2 (2 %) of 82 SCA patients. The overall ORs for *KRAS* and *GNAS* mutation in IPMN patients were 71.240 (95 % CI 16.856–301.086; *P* < 0.001, Q = 1.810, *I*^*2*^ = 0.000) and 15.297 (95 % CI 4.544–51.498; *P* < 0.001, Q = 4.525, *I*^*2*^ = 11.611), respectively.

### Age and sex

**Fig. 3:**
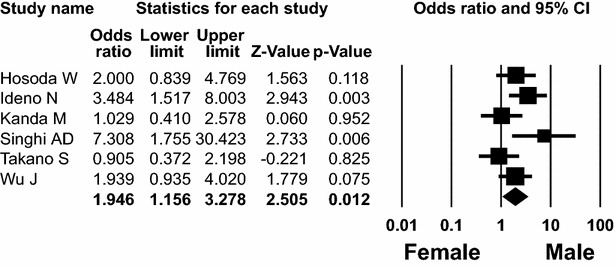
Forest plot of odds ratios with corresponding 95 % confidence intervals for the association of *GNAS* mutation of IPMN with male patients

The incidence of *KRAS* and *GNAS* mutations in patients with IPMN according to the patient’s sex was compared in eleven (Fritz et al. 2009; Hosoda et al. 2015; Kobayashi et al. 2008; Kondo et al. 1997; Mulligan et al. 1999; Schönleben et al. 2008; Singhi et al. 2014; Tada et al. 1991; Uemura et al. 2003; Wada et al. 2004; Wu et al. 2011b) and six (Hosoda et al. 2015; Ideno et al. 2015; Kanda et al. 2013; Singhi et al. 2014; Takano et al. 2014; Wu et al. 2011b) studies, respectively. *KRAS* mutation was detected in 162 (69 %) of 236 male patients and 120 (69 %) of 173 female patients with IPMN. *GNAS* mutation was detected in 198 (63 %) of 314 male patients and 110 (48 %) of 229 female patients with IPMN. The overall ORs for *KRAS* and *GNAS* mutations in male patients with IPMN were 1.065 (95 % CI 0.680–1.668; *P* = 0.782, Q = 7.216, *I*^*2*^ = 0.000) and 1.946 (95 % CI 1.156–3.278; *P* = 0.012, Q = 9.885, *I*^*2*^ = 49.419), respectively (Fig. 3).

Five (Fritz et al. 2009; Kobayashi et al. 2008; Schönleben et al. 2008; Singhi et al. 2014; Wada et al. 2004) and three (Kanda et al. 2013; Singhi et al. 2014; Wu et al. 2011b) studies presented mean age with standard deviation (SD) or p value according to *KRAS* and *GNAS* mutations, respectively. The mean age of IPMN patients with *KRAS* mutation ranged from 63.67 to 70.15 years, whereas the mean age of those with wild-type *KRAS* ranged from 64.3 to 68.58 years. The mean age of IPMN patients with *GNAS* mutation ranged from 62.14 to 69.54 years, whereas the mean age of those with wild-type *GNAS* ranged from 59 to 70.09 years. No associations were found between mean age and *KRAS* (WMD = 0.140; 95 % CI −0.194 to 0.475; *P* = 0.410, Q = 2.940, *I*^*2*^ = 0.000) or *GNAS* (WMD = 0.099; 95 % CI −0.156 to 0.354; *P* = 0.448, Q = 1.838, *I*^*2*^ = 0.000) mutations.

### Location and tumour size

The incidence of *KRAS* or *GNAS* mutation in patients with IPMN according to the location (head versus body or tail) was compared in eight (Hosoda et al. 2015; Kobayashi et al. 2008; Kondo et al. 1997; Schönleben et al. 2008; Singhi et al. 2014; Uemura et al. 2003; Wada et al. 2004; Wu et al. 2011b) and six (Hosoda et al. 2015; Ideno et al. 2015; Kanda et al. 2013; Singhi et al. 2014; Takano et al. 2014; Wu et al. 2011b) studies, respectively. *KRAS* mutation was detected in 154 (67 %) of 229 patients with IPMN arising in the pancreatic head and 97 (75 %) of 130 patients in the body or tail. *GNAS* mutation was detected in 168 (58 %) of 288 patients with IPMN arising in the pancreatic head and 125 (56 %) of 224 patients in the body or tail. There were no associations between *KRAS* or *GNAS* mutation and tumour location (OR 0.836, 95 % CI 0.477–1.465, *P* = 0.532, Q = 7.624, *I*^*2*^ = 8.182 and OR 1.133, 95 % CI 0.785–1.634, *P* = 0.505, Q = 2.131, *I*^*2*^ = 0.000, respectively).

Four (Kanda et al. 2013; Kuboki et al. 2015; Singhi et al. 2014; Wu et al. 2011b) and three (Kuboki et al. 2015; Singhi et al. 2014; Wada et al. 2004) studies presented mean tumour size with SD or p value according to *KRAS* or *GNAS* mutation, respectively. The average tumour size of IPMN patients with *KRAS* mutations ranged from 2.4 to 2.9 cm, whereas the mean size of IPMNs with wild-type *KRAS* ranged from 2.51 to 2.86 cm. The mean tumour size of IPMNs with *GNAS* mutation ranged from 1.234 to 3.859 cm, whereas the average size of IPMNs with wild-type *GNAS* ranged from 1.134 to 3.66 cm. No relationship was found between the average tumour size and *KRAS* or *GNAS* mutation (WMD = 0.000, 95 % CI −0.258 to 0.258, P > 0.999, Q = 0.860, *I*^*2*^ = 0.000 and WMD = 0.086, 95 % CI −0.108 to 0.280, P = 0.384, Q = 0.315, *I*^*2*^ = 0.000, respectively).

### Macroscopic and microscopic duct types of IPMNs

Nine (Fritz et al. 2009; Hosoda et al. 2015; Kaino et al. 1999; Kobayashi et al. 2008; Kondo et al. 1997; Kuboki et al. 2015; Lee et al. 2014; Singhi et al. 2014; Wu et al. 2011b) and seven (Hosoda et al. 2015; Ideno et al. 2015; Kuboki et al. 2015; Lee et al. 2014; Singhi et al. 2014; Takano et al. 2014; Wu et al. 2011b) studies addressed *KRAS* and *GNAS* mutations in IPMN patients, according to the macroscopic duct type, respectively (Table 4). *KRAS* mutation was found in 97 (61 %) of 160 main duct type, 191 (70 %) of 272 branch duct type, and 58 (62 %) of 94 mixed duct type. *GNAS* mutation was found in 105 (56 %) of 187 main duct type, 160 (49 %) of 327 branch duct type, and 45 (51 %) of 89 mixed duct type. *KRAS* and *GNAS* mutations were not significantly related to main (OR 0.614, 95 % CI 0.342–1.102, *P* = 0.102, Q = 10.686, *I*^*2*^ = 34.496 and OR 1.346, 95 % CI 0.934–1.939, *P* = 0.681, Q = 3.972, *I*^*2*^ = 0.000, respectively) and branch duct types (OR 1.662, 95 % CI 0.859–3.216, *P* = 0.132, Q = 13.149, *I*^*2*^ = 46.764 and OR 0.815, 95 % CI 0.585–1.136, *P* = 0.577, Q = 4.743, *I*^*2*^ = 0.000, respectively).

**Table 4:** Characteristics of individual studies of *KRAS* and *GNAS* mutations in patients with IPMN according to the macroscopic duct types

Study	*KRAS* mutation (mutation/total) (%)			*GNAS* mutation (mutation/total) (%)		
	Main duct	Branch duct	Mixed duct	Main duct	Branch duct	Mixed duct
Fritz S	2/2 (100)	0/2 (0)	6/16 (38)			
Hosada W	25/41 (61)	34/50 (68)		26/41 (63)	29/50 (58)	
Ideno N				2/6 (33)	22/45 (49)	7/11 (64)
Kaino M	5/5 (100)	7/7 (100)				
Kobayashi N	1/2 (50)	12/20 (60)				
Kondo H	3/3 (100)	9/10 (90)				
Kuboki Y	29/50 (58)	42/81 (52)	25/41 (61)	27/50 (54)	41/81 (51)	15/41 (37)
Lee LS	3/9 (33)	6/10 (60)		4/9 (44)	4/10 (40)	
Singhi AD	6/13 (46)	23/28 (82)	6/9 (67)	4/13 (31)	11/28 (39)	3/9 (33)
Takano S				18/33 (55)	16/49 (33)	
Wu J	23/35 (66)	58/64 (91)	21/28 (75)	24/35 (69)	38/64 (59)	20/28 (71)

**Fig. 4:**
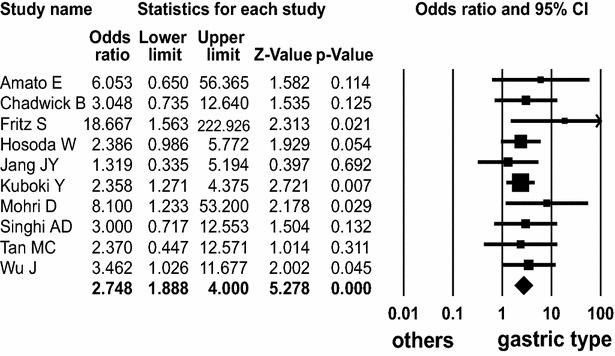
Forest plot of odds ratios with corresponding 95 % confidence intervals for the association of *KRAS* mutation with gastric subtype of IPMN

Ten (Amato et al. 2014; Chadwick et al. 2009; Fritz et al. 2009; Hosoda et al. 2015; Jang et al. 2009; Kuboki et al. 2015; Mohri et al. 2012; Singhi et al. 2014; Tan et al. 2015; Wu et al. 2011b) and six (Amato et al. 2014; Hosoda et al. 2015; Kuboki et al. 2015; Singhi et al. 2014; Tan et al. 2015; Wu et al. 2011b) studies described *KRAS* and *GNAS* mutations in IPMN patients, according to the microscopic duct type, respectively (Table 5). *KRAS* mutation was detected in 228 (73 %) of 314 gastric type, 47 (72 %) of 65 pancreatobiliary duct type, 99 (44 %) of 227 intestinal type, and 5 (29 %) of 17 oncocytic type. *KRAS* mutation was significantly found in gastric type (OR 2.748; 95 % CI 1.888–4.000; *P* < 0.001, Q = 5.679, *I*^*2*^ = 0.000) with high frequency (Fig. 4), compared to its frequency in intestinal type (OR 0.311; 95 % CI 0.206–0.471; *P* < 0.001, Q = 10.036, *I*^*2*^ = 10.324).

**Table 5:** Characteristics of individual studies of *KRAS* and *GNAS* mutations in patients with IPMN according to the microscopic duct types

Study	*KRAS* mutation (mutation/total) (%)				*GNAS* mutation (mutation/total) (%)			
	Gastric	Pancreato biliary	Intestinal	Oncocytic	Gastric	Pancreato biliary	Intestinal	Oncocytic
Amato E	5/6 (83)	3/3 (100)	14/36 (39)	2/3 (67)	6/6 (100)	1/3 (33)	30/36 (83)	1/3 (33)
Chadwick B	16/19 (84)	6/7 (86)	15/26 (58)					
Fritz S	7/10 (70)		1/7 (14)	0/2 (0)				
Hosada W	40/55 (73)	6/7 (86)	12/27 (44)	1/2 (50)	34/55 (62)	0/7 (0)	21/27 (74)	0/2 (0)
Jang JY	5/13 (38)	4/8 (50)	4/19 (21)					
Kuboki Y	63/97 (65)	7/11 (64)	25/56 (45)	1/8 (13)	45/97 (46)	3/11 (27)	33/56 (59)	1/8 (13)
Mohri D	9/11 (82)	1/1 (100)	3/11 (27)	1/2 (50)				
Singhi AD	30/40 (75)	3/5 (60)	2/5 (40)		13/40 (33)	0/5 (0)	5/5 (100)	
Tan MC	8/10 (80)	10/16 (63)	17/27 (63)		5/10 (50)	5/16 (31)	20/27 (74)	
Wu J	45/52 (87)	7/7 (100)	6/13 (46)		34/52 (65)	3/7 (43)	13/13 (100)

**Fig. 5:**
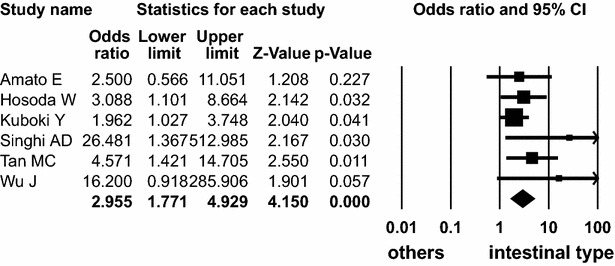
Forest plot of odds ratios with corresponding 95 % confidence intervals for the association of *GNAS* mutation with intestinal subtype of IPMN

On the other hand, *GNAS* mutation was detected in 122 (74 %) of 164 intestinal type, 137 (53 %) of 260 gastric type, 12 (24 %) of 49 pancreatobiliary duct type, and 2 (15 %) of 13 oncocytic type. *GNAS* mutation was significantly found in intestinal type with high frequency (OR 2.955; 95 % CI 1.771–4.929; *P* < 0.001, Q = 5.537, *I*^*2*^ = 9.694) (Fig. 5) and was present in pancreatobiliary and oncocytic types with low frequency (OR 0.220; 95 % CI 0.108–0.450; *P* < 0.001, Q = 3.009, *I*^*2*^ = 0.000 and OR 0.128; 95 % CI 0.031–0.537; *P* = 0.005, Q = 0.033, *I*^*2*^ = 0.000, respectively).

However, *KRAS* mutation was not significantly associated with pancreatobiliary and oncocytic types, respectively (OR 1.604, 95 % CI 0.873–2.947, *P* = 0.128, Q = 4.971, *I*^*2*^ = 0.000 and OR 0.415; 95 % CI 0.129–1.336; *P* = 0.140, Q = 3.643, *I*^*2*^ = 0.000, respectively). *GNAS* mutation was not significantly associated with gastric type (OR 0.845, 95 % CI 0.560–1.275, *P* = 0.422, Q = 3.189, *I*^*2*^ = 0.000, respectively).

### Histologic grade and presence of adenocarcinoma

**Fig. 6:**
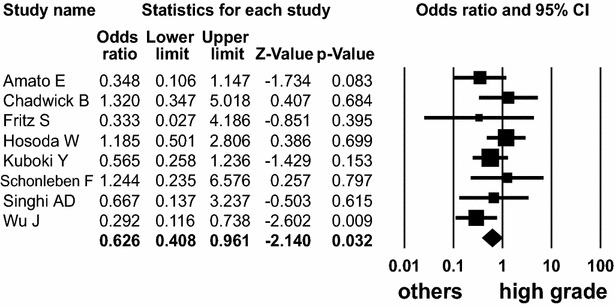
Forest plot of odds ratios with corresponding 95 % confidence intervals for the association of *KRAS* mutation with high-grade dysplasia of IPMN

Nine (Amato et al. 2014; Chadwick et al. 2009; Fritz et al. 2009; Hosoda et al. 2015; Kuboki et al. 2015; Lubezky et al. 2011; Schönleben et al. 2008; Singhi et al. 2014; Wu et al. 2011b) and seven (Amato et al. 2014; Hosoda et al. 2015; Ideno et al. 2015; Kanda et al. 2013; Kuboki et al. 2015; Singhi et al. 2014; Wu et al. 2011b) studies presented *KRAS* and *GNAS* mutations of IPMN patients, according to the histologic grades. *KRAS* mutation was detected in 132 (58 %) of 227 high grade, 95 (70 %) of 136 intermediate grade, and 129 (63 %) of 205 low grade. *KRAS* mutation was detected in high grade dysplasia with lower frequency (OR 0.626; 95 % CI 0.408–0.961; *P* = 0.032, Q = 7.797, *I*^*2*^ = 10.219) (Fig. 6).

However, the prevalence of *KRAS* mutation in low and intermediate grades was not statistically significant (OR 1.521, 95 % CI 0.984–2.353, *P* = 0.059, Q = 1.929, *I*^*2*^ = 0.000, and OR 1.139, 95 % CI 0.657–1.976, *P* = 0.643, Q = 8.326, *I*^*2*^ = 15.929, respectively). *GNAS* mutation was detected in 120 (61 %) of 198 high grade, 86 (67 %) of 128 intermediate grade, and 119 (56 %) of 213 low grade. The frequency of *GNAS* mutation did not differ among high, intermediate, and low grades of IPMN patients (OR 0.769, 95 % CI 0.382–1.547, *P* = 0.461, Q = 15.126, *I*^*2*^ = 60.334; OR 1.273, 95 % CI 0.786–2.060, *P* = 0.326, Q = 2.789, *I*^*2*^ = 0.000, and OR 0.938, 95 % CI 0.500–1.761, *P* = 0.843, Q = 12.357, *I*^*2*^ = 51.445, respectively). The subgroup analysis revealed that the detection methods influenced the relationship between *GNAS* mutation and high grade dysplasia of IPMN (Additional file 2: Table S2).

Twenty-one (Amato et al. 2014; Fritz et al. 2009; Hosoda et al. 2015; Ideno et al. 2015; Jang et al. 2009; Kondo et al. 1997; Kuboki et al. 2015; Lubezky et al. 2011; Mizuno et al. 2010; Mohri et al. 2012; Mueller et al. 2003; Mulligan et al. 1999; Nakata et al. 2002; Raimondo et al. 2002; Schönleben et al. 2008; Sessa et al. 1994; Singhi et al. 2014; Uemura et al. 2003; Wada et al. 2004; Wu et al. 2011b; Yoshizawa et al. 2002) and six (Amato et al. 2014; Hosoda et al. 2015; Ideno et al. 2015; Kuboki et al. 2015; Singhi et al. 2014; Wu et al. 2011b) studies reported *KRAS* and *GNAS* mutations in IPMN patients, according to the presence of associated pancreatic adenocarcinoma. *KRAS* mutation was present in 190 (64 %) of 297 IPMN patients with invasive adenocarcinoma, whereas 388 (63 %) of 620 IPMNs without adenocarcinoma. *GNAS* mutation was present in 80 (51 %) of 157 IPMN patients with invasive adenocarcinoma, whereas 265 (59 %) of 446 IPMNs without adenocarcinoma. No significant associations were seen between *KRAS* and *GNAS* mutations and the presence of adenocarcinoma in IPMN patients (OR 1.342, 95 % CI 0.878–2.050, *P* = 0.174, Q = 26.746, *I*^*2*^ = 25.222, and OR 0.548, 95 % CI 0.285–1.053, *P* = 0.071, Q = 10.231, *I*^*2*^ = 51.131, respectively). The subgroup analyses disclosed that the ethnicity and detection methods of these mutations did not influence the relationship between *GNAS* mutation and IPMN-associated adenocarcinoma (Additional file 2: Table S2).

### Sensitivity analysis and publication bias

The sensitivity analyses showed that none of the studies affected the pooled prevalence rate, OR, or WMD with CIs, except the pooled analysis of *GNAS* mutation between the genders and of *KRAS* mutation between high grade and the other grades of IPMNs (Additional file 3: Fig. S1). Through the funnel plots and the Egger’s regression tests, the pooled results from *KRAS* mutation between tumour locations within the pancreas, *GNAS* mutation between mean tumour size and between intermediate grade and the other grades, and *KRAS* and *GNAS* mutations between microscopic duct subtypes of IPMNs showed the possibility of publication bias. However, other pooled analyses showed no evidence of publication bias (Additional file 4: Table S3) (Additional file 5: Fig. S2).

## Discussion

This pooled analysis using data from 1253, 835, and 143 pancreatic IPMN patients revealed that overall *KRAS*, *GNAS*, and *RNF43* mutations were detected in 61, 56, and 23 %, respectively. These gene mutation rates did not differ according to the ethnicity, detection methods, and specimen type. This meta-analysis showed that the frequencies of *KRAS* and *GNAS* mutations in IPMN patients were considerably variable among microscopic duct subtypes.

The most common preoperative challenge is to distinguish IPMN from other cystic lesions of the pancreas. There are three primary types of pancreatic cystic neoplasm: IPMN, MCN, and SCA (Wu et al. 2011b). Most of the pancreatic adenocarcinomas develop from IPMNs, followed by MCNs. In contrast, SCAs do not give rise to invasive adenocarcinomas (Wu et al. 2011b). Until now, preoperative cystic fluid evaluation for CEA, amylase, DNA methylation, and microRNA expression remains suboptimal, partly because of their lack of disease specificity (Weinstein et al. 2004). Based on the high frequencies and significant ORs of *KRAS* and *GNAS* mutations in IPMNs compared to the other cystic lesions, particularly MCNs, the combined detection of *KRAS* and *GNAS* mutations from the cystic fluid would be highly valuable in the preoperative diagnosis of IPMNs.

This pooled analysis found that different mutational profile between *KRAS* and *GNAS* was significantly related to the microscopic subtypes of IPMNs, which are a determinant for the subtypes of invasive adenocarcinomas. Over 30 % of intestinal and pancreaticobiliary type IPMNs develop colloid and tubular type adenocarcinomas, respectively (Klöppel et al. 2014). In contrast, gastric type IPMNs rarely develops into invasive adenocarcinomas. When gastric type IPMNs progress to adenocarcinomas, it is the tubular type (Klöppel et al. 2014). The IPMNs with colloid adenocarcinoma is known to have a better prognosis than those with tubular adenocarcinoma (Klöppel et al. 2014; Tan et al. 2015). Colloid adenocarcinomas arising from IPMNs were associated with a high frequency of *GNAS* mutation (Tan et al. 2015). In agreement with the previous study (Tan et al. 2015), our results suggest that *KRAS* and *GNAS* mutational pattern may represent different pathways in the IPMN-adenocarcinoma sequence.

The *GNAS* gene encodes the α-subunit of the stimulatory G-protein (Gαs). Somatic activating *GNAS* mutation results in an elevated level of cyclic adenosine monophosphate (cAMP) and in uncontrolled growth signalling (Landis et al. 1989; Weinstein et al. 2004). *GNAS* mutation has been found in various tumours, fibrous dysplasia, and McCune-Albright syndrome (Landis et al. 1989; Weinstein et al. 2004). Interestingly, most of the *GNAS* mutations at codon 201 in IPMNs result in either an R201H or an R201C substitution, which are the same mutation as in endocrine neoplasms (Landis et al. 1989; Weinstein et al. 2004). The endocrine tumours with activating *GNAS* mutations have been supposed to be associated with hormonal secretion.

Recently, inactivating nonsense mutations of *RNF43* gene that encodes a protein with E3 ubiquitin ligase activity were found in IPMNs (Amato et al. 2014; Macgregor-Das and Iacobuzio-Donahue 2013; Sakamoto et al. 2015; Tan et al. 2015; Wu et al. 2011a). Our meta-analysis found that *RNF43* mutation was not associated with clinicopathologic parameters of patients with IPMN (Additional file 6: Table S4). Due to the lack of published articles, further studies need to clarify the roles and characteristics of *RNF43* mutation in IPMN patients.

This meta-analysis revealed that *KRAS* and *GNAS* mutations are not associated with the malignant potential or prognosis in patients with IPMN. This meta-analysis showed that frequency of *KRAS* mutation was rather lower in high-grade dysplasia than low- and/or intermediate-grade dysplasia. However, further more studies are needed to confirm the results. The frequency of *GNAS* mutation in IPMN patients did not differ among the three grades of dysplasia and in the absence or presence of associated adenocarcinoma. The association between *GNAS* mutation and the prognosis of patients with IPMN has been the subject of considerable controversy. *GNAS* mutation was significantly associated with high-grade dysplasia (Wu et al. 2011b), whereas wild-type *GNAS* in IPMNs was significantly related to the development of adenocarcinoma (Ideno et al. 2015). However, as with this meta-analysis, other studies failed to show significant relationships between *GNAS* mutation, dysplasia grades, and the presence of adenocarcinoma (Amato et al. 2014; Hosoda et al. 2015; Kuboki et al. 2015; Singhi et al. 2014).

It has been well known that pancreatic cancers are more frequent in Ashkenazi Jews and African groups. However, the frequency of IPMN between races has not been known because of its rare incidence. Therefore, we simply classified IPMN patients into the Caucasian and Asian groups in this study, although the genetic changes of diverse and detailed ethnicity would be an interesting issue and broaden the novel biological pathway of IPMN.

The present meta-analysis has some limitations. First, the possibility of publication bias could not be completely excluded. Second, the individual study used in this meta-analysis was done with relatively small sample sizes, due to the rare occurrence of IPMNs. Lastly, the different studies did not only use different methods for mutation detection but also different tumour materials, such as cystic fluid versus tissue specimen. This might confound the mutation detection rates.

In summary, this meta-analysis provides sensitive and specific diagnostic roles of *KRAS* and *GNAS* mutations for detecting the IPMN among the pancreatic cystic lesions. Furthermore, *KRAS* and *GNAS* mutations hint a possibility that patients with IPMN have which form of microscopic subtype.

## Additional files

10.1186/s40064-016-2847-4 Characteristics of individual studies of RNF43 mutation in IPMN.
10.1186/s40064-016-2847-4 Subgroup analysis of histologic grades and associated adenocarcinoma according to the ethnicity and detection methods in IPMN patients with GNAS mutation.
10.1186/s40064-016-2847-4 Sensitivity analysis of meta-analysis for KRAS mutation between high grade and the other low and intermediate grades of IPMN. When each study is sequentially removed and meta-analysis is repeated with the remaining studies, the pooled odd ratios remain the same.
10.1186/s40064-016-2847-4 Egger’s tests for funnel plot asymmetry.
10.1186/s40064-016-2847-4 Funnel plot for publication bias in the frequency of KRAS mutation between the main duct and branch duct of IPMN. Individual studies are represented by small circles. An area of the inverted V-shape is devoid of small negative studies, indicating publication bias.
10.1186/s40064-016-2847-4 Association between RNF43 mutation and clinicopathologic parameters of IPMN.
