# Supplementary material for: KRAS, GNAS, and RNF43 mutations in intraductal papillary mucinous neoplasm of the pancreas: a meta-analysis
Source: Springerplus. 2016 Jul 26;5(1):1172. doi: 10.1186/s40064-016-2847-4 (PMC4960083; doi:10.1186/s40064-016-2847-4)
Supplement: Supplementary file 1 — 10.1186/s40064-016-2847-4 Characteristics of individual studies of RNF43 mutation in IPMN. [file 40064_2016_2847_MOESM1_ESM.docx]

**Table S1** Characteristics of individual studies of *RNF43* mutation in IPMN

| Study | Country | Ethnicity | Detection method | Specimen | *RNF43* mutation (%) |
| --- | --- | --- | --- | --- | --- |
| Amato E | Italy | Caucasian | sequencing | tissue | 6/40 (15.0) |
| Sakamoto HJ | Japan | Asian | sequencing | tissue | 8/57 (14.0) |
| Tan MC | USA | Caucasian | sequencing | tissue | 7/38 (18.4) |
| Wu J | USA | Caucasian | exome sequencing | tissue | 6/8 (75.0) |
