# Supplementary material for: KRAS, GNAS, and RNF43 mutations in intraductal papillary mucinous neoplasm of the pancreas: a meta-analysis
Source: Springerplus. 2016 Jul 26;5(1):1172. doi: 10.1186/s40064-016-2847-4 (PMC4960083; doi:10.1186/s40064-016-2847-4)
Supplement: Supplementary file 2 — 10.1186/s40064-016-2847-4 Subgroup analysis of histologic grades and associated adenocarcinoma according to the ethnicity and detection methods in IPMN patients with GNAS mutation. [file 40064_2016_2847_MOESM2_ESM.docx]

**Table S2** Subgroup analysis of histologic grades and associated adenocarcinoma according to the ethnicity and detection methods in IPMN patients with *GNAS* mutation.

| Category | No. of studies | Odd ratio (95% CI) | *P* value |
| --- | --- | --- | --- |
| High grade dysplasia | 7 | 0.769 (0.382 – 1.547) | 0.461 |
| Ethnicity |  |  | 0.502 |
| Caucasian | 4 | 1.004 (0.351 – 2.870) |  |
| Asian | 3 | 0.620 (0.242 – 1.588) |  |
| Detection method |  |  | 0.041 |
| Sequencing | 5 | 0.592 (0.323 – 1.084) |  |
| Non-sequencing | 1 | 2.256 (0.726 – 7.003) |  |
| Low grade dysplasia | 7 | 0.938 (0.500 – 1.761) | 0.843 |
| Ethnicity |  |  | 0.090 |
| Caucasian | 4 | 0.535 (0.229 – 1.254) |  |
| Asian | 3 | 1.377 (0.695 – 2.730) |  |
| Detection method |  |  | 0.053 |
| Sequencing | 6 | 1.209 (0.698 – 2.095) |  |
| Non-sequencing | 1 | 0.350 (0.113 – 1.086) |  |
|  |  |  |  |
| Associated adenocarcinoma | 6 | 0.548 (0.285 – 1.053) | 0.071 |
| Ethnicity |  |  | 0.633 |
| Caucasian | 3 | 0.668 (0.214 – 2.082) |  |
| Asian | 3 | 0.470 (0.192 – 1.149) |  |
| Detection method |  |  | 0.054 |
| Sequencing | 5 | 0.452 (0.252 – 0.810) |  |
| Non-sequencing | 1 | 1.696 (0.506 – 5.687) |  |

CI; confidence interval
