# Supplementary material for: KRAS, GNAS, and RNF43 mutations in intraductal papillary mucinous neoplasm of the pancreas: a meta-analysis
Source: Springerplus. 2016 Jul 26;5(1):1172. doi: 10.1186/s40064-016-2847-4 (PMC4960083; doi:10.1186/s40064-016-2847-4)
Supplement: Supplementary file 4 — 10.1186/s40064-016-2847-4 Egger’s tests for funnel plot asymmetry. [file 40064_2016_2847_MOESM4_ESM.docx]

**Table S3.** Egger’s tests for funnel plot asymmetry

|  | *KRAS* | | |  | *GNAS* | |
| --- | --- | --- | --- | --- | --- | --- |
| Individual analysis | | Regression intercept | P value* |  | Regression intercept | P value* |
| IPMN vs. MCN | | 0.518 | 0.565 |  | -19.256 | 0.203 |
| IPMN vs. SCA | | -36.525 | 0.166 |  | 1.661 | 0.473 |
| Gender | | -0.283 | 0.639 |  | 2.795 | 0.442 |
| Mean age | | 0.330 | 0.909 |  | 2.479 | 0.589 |
| Location | | -1.291 | 0.080 |  | -0.242 | 0.905 |
| Mean tumour size | | 0.018 | 0.991 |  | 1.106 | 0.067 |
| Macroscopic type | |  |  |  |  |  |
| Main duct | | -0.029 | 0.977 |  | -1.287 | 0.191 |
| Branch duct | | 0.564 | 0.590 |  | -0.273 | 0.833 |
| Microscopic type | |  |  |  |  |  |
| Intestinal type | | -1.888 | 0.024 |  | 1.883 | 0.012 |
| Pancreatobiliary type | | 1.493 | 0.051 |  | -1.711 | 0.062 |
| Gastric type | | 1.162 | 0.050 |  | 0.010 | 0.991 |
| Oncocytic type | | 2.053 | 0.570 |  | -0.408 | 0.609 |
| Histologic grade of dysplasia | |  |  |  |  |  |
| High grade | | 0.174 | 0.894 |  | -0.749 | 0.683 |
| Intermediate grade | | -0.841 | 0.588 |  | 1.789 | 0.011 |
| Low grade | | -0.202 | 0.655 |  | -0.610 | 0.710 |
| Associated adenocarcinoma | | 0.312 | 0.602 |  | -1.942 | 0.290 |

*, Two-sided P value < 0.1 was statistically significant
