# Supplementary material for: KRAS, GNAS, and RNF43 mutations in intraductal papillary mucinous neoplasm of the pancreas: a meta-analysis
Source: Springerplus. 2016 Jul 26;5(1):1172. doi: 10.1186/s40064-016-2847-4 (PMC4960083; doi:10.1186/s40064-016-2847-4)
Supplement: Supplementary file 6 — 10.1186/s40064-016-2847-4 Association between RNF43 mutation and clinicopathologic parameters of IPMN. [file 40064_2016_2847_MOESM6_ESM.docx]

**Table S4** Association between *RNF43* mutation and clinicopathologic parameters of IPMN

| Category | No. of  studies | Mutation cases  / total (%) | Odd ratio (95% CI) | *P* value | Q | *I ^2^* |
| --- | --- | --- | --- | --- | --- | --- |
| Microscopic subtype |  |  |  |  |  |  |
| intestinal type | 3 | 14/76 (18.4%) | 1.323 (0.527 – 3.319) | 0.551 | 1.575 | 0.000 |
| pancreatobiliary type | 3 | 6/22 (27.3%) | 2.225 (0.715 – 7.110) | 0.165 | 1.272 | 0.000 |
| gastric type | 3 | 5/48 (10.4%) | 0.530 (0.169 – 1.667) | 0.277 | 0.164 | 0.000 |
| Histologic grade |  |  |  |  |  |  |
| low grade | 3 | 5/51 (9.8%) | 0.388 (0.125 – 1.198) | 0.100 | 0.545 | 0.000 |
| high grade | 3 | 14/69 (20.3%) | 1.669 (0.478 – 5.822) | 0.422 | 2.974 | 32.756 |
| Associated adenocarcinoma | 2 | 5/37 (13.5%) | 0.854 (0.251 – 2.902) | 0.801 | 0.016 | 0.000 |

No.; number, CI; confidence interval
